# The Risk of Orthorexia and the Prevalence of Emotional Eating Behaviours among Polish Military Flying Personnel in Relation to Body Mass Index (BMI) and Sociodemographic Factors

**DOI:** 10.3390/nu16050682

**Published:** 2024-02-28

**Authors:** Agata Gaździńska, Paweł Jagielski, Paulina Baran

**Affiliations:** 1 Laboratory of Dietetics and Obesity Treatment, Department of Psychophysiological Measurements and Human Factor Research, Military Institute of Aviation Medicine, Krasińskiego 54/56, 01-755 Warsaw, Poland; 2 Faculty of Health Science, Department of Nutrition and Drug Research, Jagiellonian University Medical College, Skawińska 8, 31-066 Cracow, Poland; paweljan.jagielski@uj.edu.pl; 3 Department of Psychophysiological Measurements and Human Factor Research, Military Institute of Aviation Medicine, Krasińskiego 54/56, 01-755 Warsaw, Poland; pbaran@wiml.waw.pl

**Keywords:** emotional eating, emotional eater questionnaire (EEQ), orthorexia, ORTO-15 questionnaire, body mass, military flying personnel, Polish army

## Abstract

Background: Proper nutrition has a positive impact on health. Paradoxically, excessive preoccupation with healthy eating may lead to the emergence of abnormal eating behaviours and increase the risk of developing disorders. The aim of this study was to assess the risk of orthorexia (ON) and the prevalence of emotional eating (EE) in military flying personnel of the Polish Air Force in relation to BMI and sociodemographic factors. Methods: This study included 760 soldiers (including 60 females) taking part in the National Health Programme 2021–2025. The ORTO-15 questionnaire and EEQ were used to assess the risk of ON and EE. Results: The risk of ON occurred in 28.9% of military flying personnel and was significantly more frequent in soldiers with a normal weight (46.4%), under 40 years of age (42.7%), and with higher education (42.7%). The prevalence of EE was found in approximately 12.3% of the respondents and was significantly more common in soldiers with diagnosed obesity (17.5%), women (21.7%), and soldiers with higher education (13.6). Other sociodemographic variables did not differentiate the results of the ORTO-15 questionnaire and EEQ. Conclusions: The obtained results indicate that the problem of eating disorders also occurs in military populations. The necessity of continuing research in this area is discussed.

## 1. Introduction

The increasingly observed effects of lifestyle diseases, in both health and social dimensions, as well as the efforts made to counteract them, have resulted in greater attention being paid to lifestyle and health-related behaviours. Nowadays, increasingly more people are suffering from diseases related to unhealthy lifestyles and stress. No one has any doubts that proper nutrition, which is an important part of a healthy lifestyle, has a positive impact on health and reduces the risk of overweight and obesity, cardiovascular diseases, diabetes and cancer [1,2].

Paradoxically, however, for some people, an excessive interest in a healthy lifestyle, including preoccupation with healthy eating, may be a source of health and social problems. A lack of distance from publicly available information on healthy eating and conflicting reports on the impact of specific food products on health, as well as pressure from the environment towards a healthy lifestyle, may lead to the appearance of incorrect eating behaviours and increase the risk of, e.g., orthorexia or overeating under the influence of emotions [3,4].

The concept of orthorexia (from the Latin orthorexia nervosa (ON)) was introduced to the literature in 1997 by the American doctor Steven Bratman, and means a pathological fixation on eating proper and healthy food [5]. The prefix ortho means “correct”, while orexis means “hunger, appetite” [6]. According to Bratman’s observations, people suffering from orthorexia avoid eating certain foods and/or certain food processing methods, e.g., frying, because they believe that they are harmful to their health. They obsessively focus on the quality and method of preparing meals, strictly following the rules regarding the appearance and composition of food, and often avoid food of specific colours which contain dyes and preservatives [7]. People suffering from orthorexia spend many hours a day carefully planning what they are going to eat. The consequence of this “obsession” with healthy eating may be an ill-balanced, deficient diet resulting from imposed restrictions and the gradual elimination of an increasingly wider group of products, but also social isolation and intolerance towards people who eat differently [8].

The primary aspiration in orthorexia is the desire to achieve optimal health through strict dietary control [9]. The motivation itself to undertake a healthy diet is usually valid, and is most often based on the desire to avoid serious illnesses, improve the physical condition, and acquire healthy eating habits. However, orthorexia does not lead to an optimal nutritional state; on the contrary, it causes a number of somatic complications and deficiencies of particular vitamins and microelements, as well as disorders of the acid–base and water–electrolyte balances in the body [10]. The literature reports serious health complications in the course of orthorexia, such as severe hyponatremia, hypokalaemia, metabolic acidosis, emphysema, and pancytopenia [11].

Researchers disagree on whether orthorexia is a new eating disorder, a variant of another currently recognised eating disorder, or a completely different disease entity. Indeed, it has features in common with obsessive compulsive disorder, eating disorders, and somatoform disorders. What orthorexia and other eating disorders have in common is the presence of features such as an excessive focus on food, restrictive dieting, perfectionism, co-occurrence of anxiety, the need for control, and rigidity of behaviour and rituals related to food preparation. It is noteworthy that although orthorexia appears in the literature on eating disorders, it is still not included in the ICD-10 or DSM-5 classifications of diseases and disorders [12].

The determinants of the occurrence of orthorexia nervosa are not fully understood, but it is indicated that psychological and socio-cultural factors play a significant role in its development [13].

An analysis of current research identifies potential factors influencing the emergence of orthorexia [14]. For example, Strahler [15] showed that ON is positively related to stress, anxiety, and depressive symptoms and negatively related to psychological well-being and life satisfaction.

Researchers disagree on whether factors such as gender or self-esteem predispose a person to orthorexia [16]. A review of psychosocial risk factors for ON by Sarah et al. [17] generally found that gender and self-esteem were not associated with ON. The aforementioned researchers showed that people who disclose a history of obsessive compulsive traits, depression, and eating disorders and/or who are preoccupied with their body image are at higher risk of developing the condition. Studies have also found that eating patterns consistent with vegetarian and vegan diets also predispose a person to the development of orthorexia [18,19]. As noted, those most at risk of developing orthorexia are lacto-vegetarians and those who follow a strict eating schedule, who spend a lot of time preparing meals.

Other individuals who may be susceptible to the development of this disorder are those who undertake intense physical activity, have an increased BMI, have a higher education, or suffer from other eating disorders and/or obsessive compulsive disorders [20,21]. The prevailing fashion for both thinness and a muscular body may also be conducive to orthorectic behaviour [22,23].

Military flying personnel, due to the nature of their work and duties, should be characterised by good health, which is achieved and maintained, among other things, through proper nutrition. Hence, paying attention to the daily diet should be particularly important for this professional group. However, it is worrying that approximately 30% of cadets of the Polish Air Force Military Academy are characterised by an increased body weight [24]. In fact, the prevalence of overweight and obesity in Polish military flying personnel, depending on the adopted assessment criterion, was found to be 63.5% of soldiers according to BMI and 52.5% according to the percentage of body fat [25].

It appears that one of the main factors in the development of obesity among Polish soldiers is an increase in food intake when feeling stressed, when under tension, or when experiencing a drop in mood [26], which is a characteristic of so-called emotional eating.

Emotional eating (EE) is defined as the tendency to overeat as a regulation mechanism, i.e., coping with negative emotions, depression, anxiety, and stress [27]. EE is characterised by an inability to distinguish between the physiological sensation of hunger and the desire to use food as a strategy to cope with negative emotions. People who eat under the influence of emotions tend to overeat when they feel anxious, stressed, sad, angry, or under pressure from their environment [28].

In the general population, overweight and obesity are not uncommonly associated with emotional eating behaviour, i.e., eating in response to stress experienced. In contrast, in soldiers, a population with unique stressors, the prevalence of this phenomenon has not been studied in depth. And while mental health disorders, such as depression and post-traumatic stress disorder, are relatively well recognised in the military community, the prevalence of eating disorders, although increasingly diagnosed in this population, remains difficult to assess. The specific nature of military service, including the discipline involved, the hierarchical structure and subordination associated with ranks, the need to work as a team under varied conditions, including high stress, the separation from loved ones, and the risks associated with participation in combat situations, can lead to the use of harmful coping mechanisms to deal with tension and stress. One of these may be inappropriate eating behaviours that increase the risk of developing an eating disorder.

The incidence of ON varies significantly depending on the population studied. So far, one of the most frequently used methods for assessing ON in research is the ORTO-15 questionnaire [29]. Research conducted around the world indicates that the incidence of ON in the general population ranges from less than 1% to 58%, while in populations within the so-called risk groups, the incidence of ON can reach up to 90% [30]. To our knowledge, no one has performed such an assessment of Polish Army soldiers, including military flying personnel.

Therefore, the aim of this study is to assess the incidence of orthorexic behaviour and emotional eating among Polish military flying personnel depending on BMI and selected socio-demographic factors.

## 2. Materials and Methods

### 2.1. Study Population

This study included 760 soldiers (700 males and 60 females), members of the active military flying personnel of the Polish Air Force, who took part in the National Health Programme 2021–2025. Females accounted for 7.9% of the subjects, reflecting the general frequency of the percentage of females in the general population of the Polish Army. The mean age of the subjects was 39.39 ± 8.17 years.

The study group included individuals who consecutively reported for mandatory annual anthropometric examinations to the Laboratory of Dietetics and Obesity Treatment as part of routine adjudicatory and medical examinations at the Military Institute of Aviation Medicine in Warsaw, Poland. They expressed a desire to participate in additional examinations carried out as part of the National Health Program 2021–2025. All of them had current fitness to fly certificates given by Aeromedical Board (i.e., they were healthy). All procedures were conducted in January–December 2023. The research was approved by the Institutional Review Board of the Military Institute of Aviation Medicine, Warsaw, Poland (decision No. 01/2018 of 9 March 2018). All participants signed informed consent forms.

### 2.2. Applied Questionnaires

I. This study used a self-designed sociodemographic questionnaire, collecting such information about the subjects as age, gender, marital status, education, places of residence, and data on who they live with on a daily basis (husband/wife, children, other family members, roommate, alone).
II. The study used the ORTO-15 Questionnaire by L.M. Donini [29], adapted to Polish by Stochel et al. [31], to assess the risk of orthorexic behaviour. The ORTO-15 questionnaire consists of 15 questions regarding the obsessive approach to selecting, buying, preparing, and eating meals considered healthy, rated on a scale of “always”, “often”, “sometimes”, or “never”. The questions focus on the cognitive area of the disorder and clinical and emotional aspects. The range of possible results in the questionnaire is from 15 to 60 points. A lower score indicates a higher risk of orthorexia. The cut-off threshold for diagnosing the risk of orthorexia was set at 35 points, in accordance with the research conducted by Stochel et al. [31].
III. The Emotional Eater Questionnaire (EEQ) developed and validated by Garaulet [32], adapted to Polish by Skolmowska et al. [33], was used to assess emotional eating among military flying personnel.

The questionnaire consists of 10 questions relating to various eating behaviours, which are rated on a four-point scale, i.e., “never”, sometimes”, “generally”, and “always”. A maximum of 30 points can be obtained in the EEQ. A higher score indicates more healthy eating behaviour.

### 2.3. Assessment of Nutritional Status

Body height was measured with an anthropometer (Holtain, Crosswell, UK, https://holtain.co.uk/anth.php (accessed on 15 January 2024)) to the nearest 1 mm in a standing upright position without shoes. Body mass was determined in underwear alone, after emptying the bladder. Participants were categorized on the basis of body mass index (BMI) based on World Health Organisation (WHO) criteria [34], according to which, a BMI in the range of 18.5–24.9 is normal, a BMI in the range of 25.0–29.9 is overweight, and a BMI ≥ 30.0 indicates obesity.

### 2.4. Statistical Analysis

For quantitative variables, the mean value, standard deviation, median, first quartile (Q1), third quartile (Q3), minimum and maximum were calculated. To check for differences between groups for qualitative variables, the chi-square test was used. A correlation analysis between quantitative variables was performed using Spearman’s rank correlation coefficient. In order to analyse the relationship between age and the risk of orthorexia and emotional eating, the subjects were additionally divided into 2 age groups: <40 and ≥40 years. The analyses were performed in the PS IMAGO PRO 9 program (IBM SPSS Statistics 29); the level of statistical significance was assumed to be *p* < 0.05.

## 3. Results

The characteristics of the surveyed group of Polish military flying personnel are presented in Table 1.

**Figure 1:**
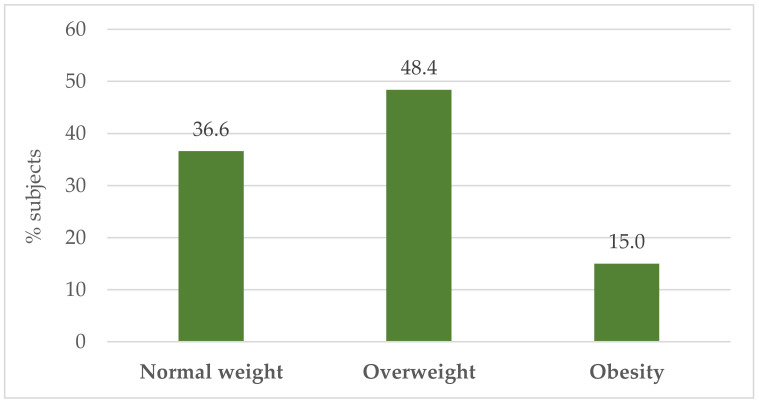
Percentage prevalence of overweight and obesity in surveyed soldiers according to BMI.

As can be seen, the youngest respondent was 19, while the oldest was 60 years old. The average BMI of the soldiers was 26.58 ± 3.80 kg/m^2^. A total of 36.6% of the respondents had a normal body weight, while the largest number of people, i.e., 48.4% were diagnosed as overweight (see Figure 1).

**Figure 2:**
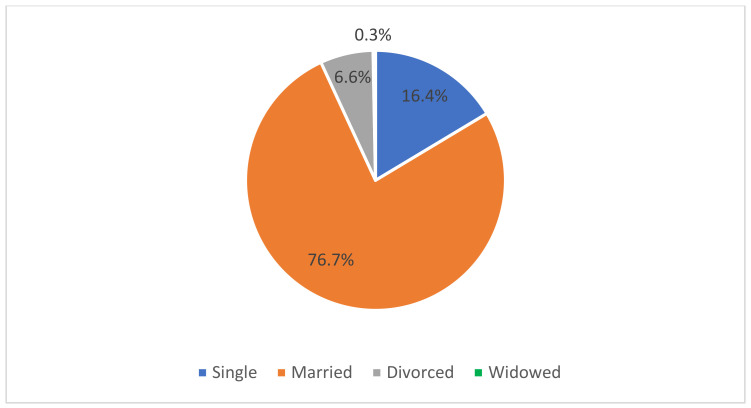
Marital status of respondents.

The survey results showed that the majority of respondents, i.e., 76.7%, were married (see Figure 2). A total of 83.6% of respondents had higher education and 46.6% of soldiers declared that they lived in the countryside and in small towns (<50,000 inhabitants). The remaining respondents lived in large cities with over 50,000 inhabitants. When asked who they lived with on a daily basis, only 11.4% of all respondents answered that they currently live alone.

**Figure 3:**
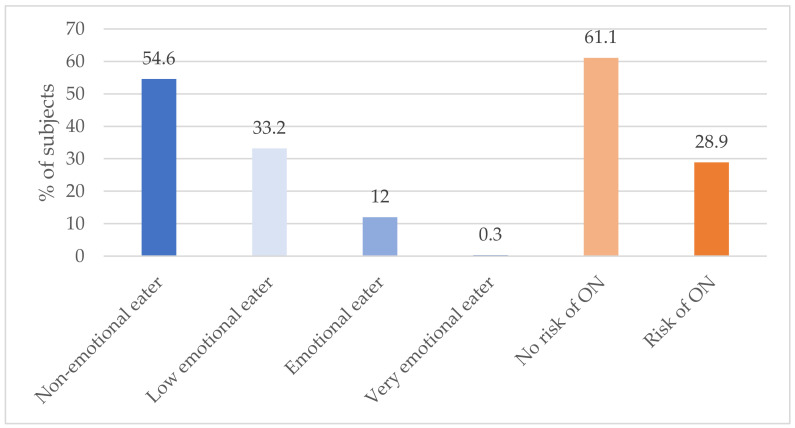
Results of the EEQ and ORTO-15 questionnaire. ON—orthorexia nervosa.

The average result obtained in the ORTO-15 questionnaire over the entire study population was 36.45 ± 3.95 points (see Table 1). The study revealed that 28.9% of the military flying personnel were at risk of developing orthorexia (see Figure 3).

The average score obtained in the EEQ regarding emotional eating in the group of surveyed soldiers was 5.84 ± 3.80 points (see Table 1). More than half of the respondents (415 people) showed no tendency to eat emotionally, 12% of the respondents showed emotional eating (91 people), while only 2 people (0.3%) reported that their eating habits were strongly dependent on emotional factors (see Figure 3). Based on the results obtained, it can be concluded that the incidence of emotional eating in the group of military flying personnel concerns a total of 12.3% of the respondents.

**Figure 4:**
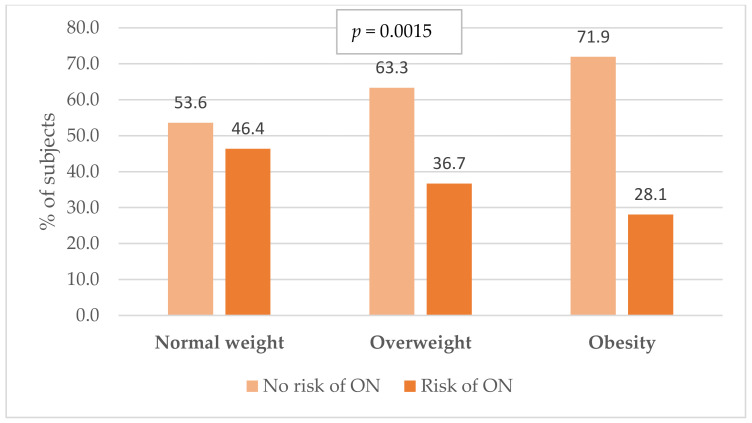
Incidence of orthorexia risk in military flying personnel in relation to body mass index (BMI). ON—orthorexia nervosa; *p*-value for the chi^2^ test.

One of the main objectives of this study was to assess the incidence of orthorexic behaviour in Polish military flying personnel depending on BMI and sociodemographic factors. The analysis of the test results showed that people with a normal body weight (i.e., a BMI in the range of 18.5–24.9 kg/m^2^) significantly more often showed orthorexic behaviour compared to people diagnosed with overweight or obesity (*p* < 0.001). The risk of orthorexia occurred in 46.4% of flight crew members with a normal body weight (see Figure 4).

The study determined whether gender, marital status, education level, place of residence, and who the person lives with on a daily basis differentiates the risk of orthorexia (see Table 2).

It was shown that the level of education is a significant factor differentiating the risk of orthorexia; namely, soldiers with a higher education were significantly more likely to exhibit orthorexic behaviour (*p* < 0.001).

The study also found a higher risk of orthorexia among military flying personnel under the age of 40 (42.7%) compared to older people (34.1% risk of orthorexia).

**Figure 5:**
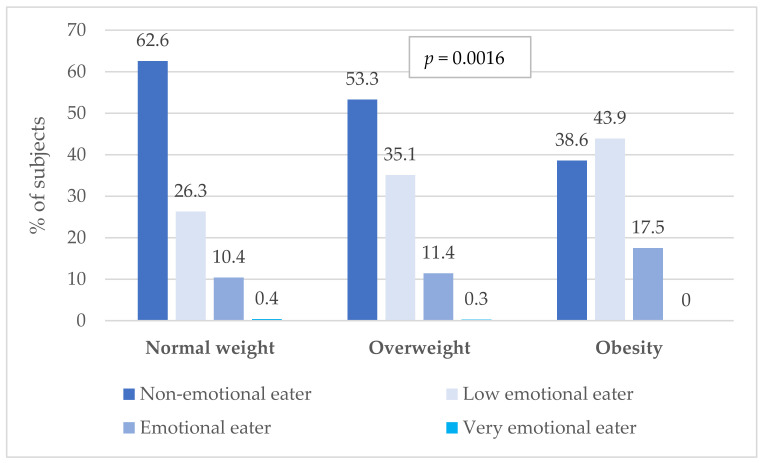
Emotional eating scores in relation to body mass index (BMI). *p*—*p*-value for the chi^2^ test.

The frequency of emotional eating, assessed on the basis of EEQ results, in relation to the BMI index in the study population is presented in Figure 5.

As can be seen in Figure 5, the study observed that people diagnosed with obesity, compared to people with a normal body weight, were characterized by significantly higher results in the EEQ (*p* < 0.001), which shows that they are significantly more susceptible to abnormal eating behaviours under the influence of emotions. The highest percentage of people eating emotionally occurred in the group of obese soldiers (17.5%), while the smallest percentage occurred in respondents with a normal body weight (10.4%). Also, the lack of symptoms indicating emotional eating was highest among flying crew members with a normal body weight (62.6%).

Significantly higher results in the EEQ were also achieved by women (21.7%) and people that were highly educated (13.6%) (*p* < 0.05) (see Table 3). The remaining sociodemographic variables did not differentiate the results of the EEQ.

**Figure 6:**
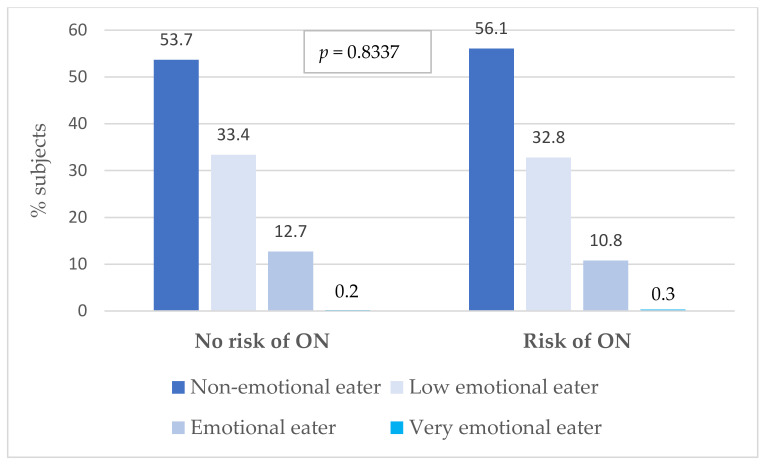
Comparison of the incidence of emotional eating in a group of subjects with a risk of ON and those with no risk of ON. ON—orthorexia nervosa; *p*-value for the chi^2^ test.

The study also examined whether military flying personnel at risk of developing orthorexia were more likely to exhibit behaviours typical of emotional eating (see Figure 6). As can be seen in the above chart, the occurrence of emotional eating in the group of people with a diagnosed risk of ON and in the group of people with no risk of developing this disorder was at a similar level and amounted to 10.8% and 12.7%, respectively (*p* > 0.05). Therefore, in the study group, no relationship between ON and EE was found.

## 4. Discussion

The number of available scientific studies on the prevalence of orthorectic behaviour and emotional eating among soldiers is still very limited, the results they provide are not consistent with each other, and, finally, many data are missing. The aim of the presented study was to assess the prevalence of orthorectic behaviour and emotional eating among members of Polish military flying personnel in relation to body mass index and selected sociodemographic factors.

The results indicate the occurrence of orthorexia risk in 28.9% of military flying personnel and emotional eating in approximately 12.3%. Regarding the risk of ON, similar results were provided by studies conducted in a group of Polish students and employees of the Faculty of Health Sciences [35,36]. On the other hand, the prevalence obtained in a study of military flight personnel was significantly higher than the prevalence determined by Donini [29], who was the first to look at the prevalence of orthorexia and estimated to be at 6.9% in the adult population (*n* = 404). In a review of the literature on prevalence by Verga et al. [37], risk groups and risk factors for the eating disorder orthorexia nervosa showed that the mean prevalence of orthorexia was also 6.9% in the general population and 35–57.8% in high-risk groups, namely healthcare professionals and artists. Some recent studies conducted in different countries indicate a prevalence rate of 21% or less using the ORTO-15 questionnaire and a cut-off point of 35 points, similar to our study [38,39,40].

In the Polish population, the prevalence of ON risk ranged from 27% to 69% using the ORTO-15 test [29,41]. Dunn and Bratman [8] suggest that the problem of orthorexia may affect up to 90% of members of certain social groups. The authors point out that the phenomenon is more prevalent in people with high levels of physical activity, as well as in those who strive to achieve the ‘ideal’ body shape that is promoted by the mass media. The results obtained in a study by Grajek et al. [42] indicate that orthorexia is three times more common in people who have theoretical knowledge and practical skills in the principles of rational nutrition and who are physically active. Given the above, it can be assumed that a higher nutritional awareness and the pursuit of high physical fitness by flight personnel are related to the higher prevalence of orthorexia risk in our study than in the adult population.

Furthermore, as the analysis of our own study results showed, the prevalence of orthorexia risk was significantly higher in those characterised by a normal weight compared to soldiers diagnosed as overweight or obese. However, different conclusions were reached by Oberle et al. [43] and Lucka et al. [44] in a study of students, in which a higher BMI was associated with a higher risk of orthorexia. This was not confirmed by the Brazilian studies of Pontes and Souza [45] and Rodrigues [46], who reported no significant correlation between orthorexia and body mass index (BMI).

A systematic review of studies [47] revealed that orthorexic tendencies were similar in both sexes, although the results differed depending on the diagnostic tool used. Some studies found that the prevalence of ON was significantly higher in women [48,49], some studies found that it was significantly higher in men [9,10], and other studies found no significant gender differences in ON [22,23,43,50,51]. In the present study, no differences were found between men and women with regard to orthorectic behaviour. Given the inconsistent results, further research is needed to clarify any potential gender differences in orthorexia risk.

In terms of emotional eating, people who have an unhealthy lifestyle, i.e., do not eat properly, move little, and cope less well with stress, are twice as likely to suffer from this disorder. Previous research suggests that the prevalence of emotionally driven overeating disorders may be lower in active service members compared to civilian populations [52].

The results we presented here allow us to conclude that military flying personnel who suffer from obesity are significantly more likely to show a tendency towards emotional eating compared to normal weight individuals. It may be that emotional eating is the cause of excessive body weight in these individuals, which would require further research. This result is consistent with a study by Janei et al. [53] which was conducted among US Army soldiers. This result is of concern because engaging in emotionally driven eating behaviours increases the risk of, among other things, the occurrence of episodes of overeating, which further hinders weight reduction and maintenance. This is supported by studies in civilian populations, which have shown that individuals overeat to reduce stress, anxiety, fear, or negative emotions, and that eating in response to emotional stimuli is associated with weight gain [54]. Therefore, it seems reasonable that soldiers suffering from obesity should receive not only dietary but also psychological support to be able to identify their own emotions and deal with them in a way that is appropriate and conducive to health. The result confirms that only a holistic approach in the treatment of obesity, involving specialists from different disciplines, is able to achieve the desired therapeutic effects.

This study found that the education level was a significant differentiating factor in the risk of both orthorexia and emotional eating. Soldiers with a higher education level scored higher in the ORTO-15 questionnaire and EEQ. Due to the self-reporting nature of the tools used, it is possible that this result is related to a higher awareness of the behaviours undertaken on a daily basis, including a higher frequency of perceiving problematic eating behaviours and consequently declaring them. This result, however, was not confirmed by a study by Dittfeld et al. [18], which showed that a kind of obsession with healthy eating characterises people with lower education to a higher degree.

The prevalence of emotional eating in the group with and without an identified ON risk was comparable. In contrast, this study found a higher risk of orthorexia in military flight attendants who were under 40 years of age compared to those who were older. Although Arusoglu et al. did not find an association between orthorexic behaviour and the age of respondents [48], Dittfeld et al. [18] in a study of vegetarians (*n* = 1346) and non-vegetarians (*n* = 1265) found a decrease in the risk of developing orthorexia with age. This result is consistent with that obtained in the present study.

Significant correlations were also found between the gender of the soldiers studied and the prevalence of emotional eating. Women, compared to men, were significantly more likely to engage in emotionally driven eating behaviours. Relating this result to other work in this area, one can cite a study by Jayne et al. [53], conducted among US Army soldiers, in which gender did not prove to be a statistically significant moderator of the association between stress and emotional eating, although the BMI significantly increased with the frequency of reported emotional eating behaviours (*p* < 0.001). Another study of US military veterans found that more women than men (32.8 percent vs. 18.8 percent, *p* < 0.001) reported symptoms specific to eating disorders included in the DSM-5 classification [55]. The estimated prevalence of each diagnosis was for men and women, respectively, anorexia nervosa (AN; 0.0% vs. 0.0%), bulimia nervosa (BN; 6.1% vs. 3.5%), binge-eating disorder (BED; 4.4% vs. 2.9%), atypical AN (AAN; 13.6% vs. 4.9%), subclinical BN (0.0% vs. 0.2%), subclinical BED (1.4% vs. 0.6%), purging disorder (2.1% vs. 0.7%), and night eating syndrome (NES; 5.2% vs. 6.0%). This study found that women were more likely to have BN and AAN. Furthermore, a study by Cole et al. [56] among active-duty military service members (*n* = 295) recruited from Texas and Washington found that men, in turn, ate more for physical rather than emotional reasons (*p* = 0.014).

Other sociodemographic variables did not differentiate the results of the ORTO-15 questionnaire and EEQ in this occupational group. The present study attempted to investigate differences in the prevalence of orthorexia risk and emotional eating according to the place of residence. The results presented here show that there were no differences in this regard, as in other studies [44]. The lack of significant differences may probably be due to the similar lifestyles of residents of both large cities and smaller towns today, where the primary source of role models relating to both lifestyle and attractiveness is the mass media, including primarily websites and portals with unrestricted access regardless of the place of residence.

This paper presents the results of the first study to assess the prevalence of orthorexia and emotional eating among Polish military flying personnel. The strengths of this paper are the large size of the study group and the pioneering nature of the study. Given the discrepancies obtained with the results of other authors, there is a need for further research on eating disorders, including orthorexic behaviour and emotional eating, occurring in military flying personnel.

## 5. Limitations

A limitation of the presented study is that the estimation of the prevalence of symptoms characteristic of orthorexia and emotional eating is based on self-reports. This means that participants may have given responses that they perceived as socially desirable, particularly given the context of them being military personnel. As a result, this may have affected the accuracy of the reported prevalence rates of orthorexia and emotional eating. However, this is a limitation of the use of questionnaire methods and should be kept in mind when interpreting the results, which may be over- or underestimated, and generalising them to the whole population.

Another limitation is that the survey was conducted among military flying personnel of the Polish Air Force, which may limit the generalisability of the results to other populations, such as civilians or soldiers from other types of armed forces.

## 6. Conclusions

The prevalence of orthorexic behaviour in the military flying personnel population was found to be 28.9%, while the prevalence of emotional eating was found to be 12.3%.

The BMI and education level of the surveyed soldiers were shown to significantly differentiate the risk of orthorexia and emotional eating. Those with a normal body weight and higher education level were significantly more likely to be at risk of orthorexia. Emotional eating, on the other hand, was significantly more frequently observed in those suffering from obesity, in those with a higher education level, and in the female group.

A higher risk of orthorexia was found in people under 40 years of age compared to older people. Other sociodemographic variables did not differentiate the risk of orthorexia and emotional eating in military flying personnel.

The research results presented in this paper on the risk of orthorexia and the frequency of emotionally driven eating behaviours show that the problem of eating disorders is real and does not bypass members of the military flying personnel of the Polish Armed Forces. Due to the inconsistency of data available in the literature, this research should be continued in order to both estimate the scale of the eating disorder phenomenon in the military environment and build psychodietetic programmes dedicated especially to soldiers at risk of eating disorders. Only an interdisciplinary approach to the treatment of eating disorders, i.e., combining specialists from various fields, including doctors, psychologists, and dieticians, will make it possible to counteract inappropriate eating behaviours and effectively deal with excessive body weight in soldiers, which is known to have a negative impact on their psychophysical performance and effects performance in combat tasks.

## Figures and Tables

**Table 1:** Characteristics of the study group (*n* = 760).

Variables	N	Mean ± SD	Median	Q1—Q3	Min–Max
Age [years]	760	39.39 ± 8.17	38.50	33.00–45.50	19.00–60.00
Weight [kg]	760	84.04 ± 13.40	83.55	75.00–92.00	50.00–182.00
Height [cm]	760	177.68 ± 7.05	178.00	173.00–182.00	149.00–199.50
BMI (kg/m^2^)	760	26.58 ± 3.80	26.03	24.08–28.72	18.21–54.05
EEQ test results [points]	760	5.84 ± 3.80	5.00	3.00–8.00	0.00–27.00
ORTO-15 test results [points]	760	36.45 ± 3.95	37.00	34.00–39.00	23.00–49.00

N: group size; SD—standard deviation; Q1—first quartile; Q3—third quartile; Min—minimum; Max—maximum.

**Table 2:** Prevalence of orthorexia risk in relation to sociodemographic factors.

No Risk or Risk of ON	Sociodemographic Factor		*p* *
Gender			
	Male (*n* = 700)	Female (*n* = 60)	
No risk of ON	61.6%	55.0%	0.3164
Risk of ON	35.4%	45.0%	
Age			
	<40 (*n* = 426)	≥40 (*n* = 334)	
No risk of ON	57.3%	65.9%	**0.0159**
Risk of ON	42.7%	34.1%	
Education			
	Higher education (*n* = 635)	Secondary education (*n* = 125)	
No risk of ON	58.4%	74.4%	**<0.001**
Risk of ON	41.6%	25.6%	
Marital status			
	Married (*n* = 583)	Others (unmarried, divorced, widowed) (*n* = 177)	
No risk of ON	61.6%	59.3%	0.5898
Risk of ON	38.4%	40.7%	
Place of residence			
	Village or city < 50,000 (*n* = 355)	City > 50,000 (*n* = 405)	
No risk of ON	62.7%	59.5%	0.3664
Risk of ON	37.3%	40.5%	
Sharing a flat with someone/living alone			
	Living with husband/wife/partner/children/roommate (*n* = 673)	Living alone (*n* = 87)	
No risk of ON	62.1%	52.9%	0.0964
Risk of ON	37.9%	47.1%	

ON—orthorexia nervosa; * *p*-value for the chi^2^ test. Bold values denote statistical significance at the *p* < 0.05 level.

**Table 3:** Results of the EEQ in relation to sociodemographic factors.

Emotional Eating Score	Sociodemographic Factor		*p* *
Gender			
	Male (*n* = 700)	Female (*n* = 60)	
Non-emotional eater	55.9%	40.0%	**0.005**
Low emotional eater	32.9%	36.7%	
Emotional eater	11.1%	21.7%	
Very emotional eater	0.1%	1.7%	
Age			
	<40 (*n* = 426)	≥40 (*n* = 334)	
Non-emotional eater	55.4%	53.6	0.2598
Low emotional eater	30.8%	36.2%	
Emotional eater	13.6%	9.9%	
Very emotional eater	0.2%	0.3%	
Education			
	Higher education (*n* = 635)	Secondary education (*n* = 125)	
Non-emotional eater	53.2%	61.6%	**0.024**
Low emotional eater	33.2%	32.8%	
Emotional eater	13.4%	4.8%	
Very emotional eater	0.2%	0.8%	
Marital status			
	Married (*n* = 583)	Others (unmarried, divorced, widowed) (*n* = 177)	
Non-emotional eater	52.8%	60.5%	0.1883
Low emotional eater	35.0%	27.1%	
Emotional eater	12.0%	11.9%	
Very emotional eater	0.2%	0.6%	
Place of residence			
	Village or city < 50,000 (*n* = 355)	City > 50,000 (*n* = 405)	
Non-emotional eater	53.1%	55.8%	0.1526
Low emotional eater	35.9%	30.9%	
Emotional eater	10.5%	13.3%	
Very emotional eater	0.6%	0%	
Sharing a flat with someone/living alone			
	Living with husband/wife/partner/children/roommate (*n* = 673)	Living alone (*n* = 87)	
Non-emotional eater	54.1%	58.6%	0.4557
Low emotional eater	34.0%	26.4%	
Emotional eater	11.6%	14.9%	
Very emotional eater	0.3%	0%	

* *p*-value for the chi^2^ test. Bold values denote statistical significance at the *p* < 0.05 level.

## Data Availability

The data is not publicly available due to the fact that it concerns soldiers of the Polish Army.
